# *Haslea silbo*, A Novel Cosmopolitan Species of Blue Diatoms

**DOI:** 10.3390/biology10040328

**Published:** 2021-04-14

**Authors:** Romain Gastineau, Gert Hansen, Michel Poulin, Claude Lemieux, Monique Turmel, Jean-François Bardeau, Vincent Leignel, Yann Hardivillier, Michèle Morançais, Joël Fleurence, Pierre Gaudin, Vona Méléder, Eileen J. Cox, Nikolaï A. Davidovich, Olga I. Davidovich, Andrzej Witkowski, Irena Kaczmarska, James M. Ehrman, Emilio Soler Onís, Antera Martel Quintana, Maja Mucko, Solenn Mordret, Diana Sarno, Boris Jacquette, Charlotte Falaise, Julie Séveno, Niels L. Lindquist, Philip S. Kemp, Elif Eker-Develi, Merve Konucu, Jean-Luc Mouget

**Affiliations:** 1 Institute of Marine and Environmental Sciences, University of Szczecin, Mickiewicza 16a, 70-383 Szczecin, Poland; nickolaid@mail.ru (N.A.D.); Andrzej.Witkowski@usz.edu.pl (A.W.); 2 Department of Biology, University of Copenhagen, Universitetsparken 4, 2100 Copenhagen, Denmark; gerth@bio.ku.dk; 3 Research and Collections, Canadian Museum of Nature, P.O. Box 3443, Station D, Ottawa, ON K1P 6P4, Canada; mpoulin@nature.ca; 4 Département de biochimie, de microbiologie et de Bio-Informatique, Institut de Biologie Intégrative et des Systèmes, Université Laval, Québec, QC G1V 0A6, Canada; claude.lemieux@bcm.ulaval.ca (C.L.); monique.turmel@bcm.ulaval.ca (M.T.); 5 Institut des Molécules et Matériaux du Mans (IMMM UMR 6283), Le Mans Université, Avenue Olivier Messiaen, CEDEX 9, 72085 Le Mans, France; Jean-Francois.Bardeau@univ-lemans.fr (J.-F.B.); Boris.Jacquette@univ-lemans.fr (B.J.); 6 FR CNRS 3473 IUML, Mer-Molécules-Santé (MMS, EA 2160), Le Mans Université, Avenue Olivier Messiaen, CEDEX 9, 72085 Le Mans, France; Vincent.Leignel@univ-lemans.fr (V.L.); yann.hardivillier@univ-lemans.fr (Y.H.); charlotte.falaise@gmail.com (C.F.); julie.sev@hotmail.fr (J.S.); Jean-Luc.Mouget@univ-lemans.fr (J.-L.M.); 7 FR CNRS 3473 IUML, Mer-Molécules-Santé (MMS, EA 2160), Université de Nantes, 2 rue de la Houssinière, CEDEX 3, 44322 Nantes, France; Michele.Morancais@univ-nantes.fr (M.M.); joel.fleurence@univ-nantes.fr (J.F.); vona.meleder@univ-nantes.fr (V.M.); 8 UMR 6112 CNRS LPG, Laboratoire de Planétologie et Géosciences, Nantes Université, 2 rue de la Houssinière, CEDEX 3, 44322 Nantes, France; pierre.gaudin@univ-nantes.fr; 9 The Natural History Museum, Cromwell Road, London SW7 5BD, UK; e.cox@nhm.ac.uk; 10 Karadag Scientific Station–Natural Reserve of the Russian Academy of Sciences, p/o Kurortnoe, Feodosiya, 98188 Crimea, Russia; olivdav@mail.ru; 11 Department of Biology, Mount Allison University, Sackville, NB E4L 1G7, Canada; iehrman@mta.ca; 12 Digital Microscopy Facility, Mount Allison University, Sackville, NB E4L 1G7, Canada; jehrman@mta.ca; 13 Observatorio Canario de Algas Nocivas (OCHABs), Parque Científico Tecnólogico Marino de Taliarte (FPCT-ULPGC), c/ Miramar, 121 Taliarte, 35214 Las Palmas, Canary Islands, Spain; esoler@marinebiotechnology.org; 14 Banco Español de Algas (BEA), Instituto de Oceanografía y Cambio Global (IOCAG), Universidad de Las Palmas de Gran Canaria (ULPGC), Muelle de Taliarte s/n, 35214 Telde, Islas Canarias, Spain; amartel@marinebiotechnology.org; 15 Faculty of Science, Biology Department, University of Zagreb, Rooseveltov trg 6, 10000 Zagreb, Croatia; maja.mucko@biol.pmf.hr; 16 Department of Research Infrastructure for Marine Biological Resources, Stazione Zoologica Anton Dohrn, 80121 Naples, Italy; solenn.mordret@szn.it (S.M.); diana.sarno@szn.it (D.S.); 17 Institute of Marine Sciences, University of North Carolina, Chapel Hill, Morehead City, NC 28557, USA; nlindquist@unc.edu; 18 Kemp Fisheries LLC, 2333 Shore Drive, Morehead City, NC 28557, USA; pskemp2@gmail.com; 19 Institute of Graduate Studies in Science, Department of Biotechnology, Mersin University, Ciftlikkoy, Mersin 33343, Turkey; elif.eker@mersin.edu.tr (E.E.-D.); mervekonucu@hotmail.com (M.K.); 20 BW24-Department of Green Chemistry and Technology, Ghent University, Coupure Links 653, B9000 Gent, Belgium

**Keywords:** Bacillariophyta, blue diatoms, *Haslea*, marennine-like pigment, new species, auxosporulation, organellar genomes, taxonomy, pseudogene

## Abstract

**Simple Summary:**

Diatoms are microalgae known for their ecological importance. Among them, just a few species are able to produce a blue pigment. We describe *Haslea silbo* sp. nov., a cosmopolitan species of blue diatoms, found on both sides of the Atlantic Ocean. The description includes the use of both microscopy and next generation sequencing. It has been possible to observe its reproduction in the laboratory, and the blue pigment it produces has also been studied.

**Abstract:**

Specimens of a new species of blue diatoms from the genus *Haslea* Simonsen were discovered in geographically distant sampling sites, first in the Canary Archipelago, then North Carolina, Gulf of Naples, the Croatian South Adriatic Sea, and Turkish coast of the Eastern Mediterranean Sea. An exhaustive characterization of these specimens, using a combined morphological and genomic approach led to the conclusion that they belong to a single new to science cosmopolitan species, *Haslea silbo* sp. nov. A preliminary characterization of its blue pigment shows similarities to marennine produced by *Haslea ostrearia*, as evidenced by UV–visible spectrophotometry and Raman spectrometry. Life cycle stages including auxosporulation were also observed, providing data on the cardinal points of this species. For the two most geographically distant populations (North Carolina and East Mediterranean), complete mitochondrial and plastid genomes were sequenced. The mitogenomes of both strains share a rare *atp6* pseudogene, but the number, nature, and positions of the group II introns inside its *cox1* gene differ between the two populations. There are also two pairs of genes fused in single ORFs. The plastid genomes are characterized by large regions of recombination with plasmid DNA, which are in both cases located between the *ycf35* and *psbA* genes, but whose content differs between the strains. The two sequenced strains hosts three plasmids coding for putative serine recombinase protein whose sequences are compared, and four out of six of these plasmids were highly conserved.

## 1. Introduction

Diatoms able to synthetize blue marennine-like pigments are restricted to a few species belonging to the genus *Haslea*, the most famous being *H. ostrearia* (Gaillon) Simonsen [1,2]. The naming of blue diatoms or blue *Haslea* comes from their remarkable ability to synthesize water soluble, blue-green, non-photosynthetic pigments, commonly called marennine-like pigments [3]. Marennine is responsible for the greening of oyster gills in Western Europe [4,5,6,7]. Marennine and marennine-like pigments also display several biological activities (e.g., antibacterial, antiviral, or antioxidant) qualifying them for putative use in aquaculture and biotechnology [8,9,10,11,12,13,14,15,16,17,18].

Since its initial description as *Vibrio ostrearius* Gaillon (1820), then *Navicula ostrearia* (Gaillon) Bory (1824), *H. ostrearia* was believed to be the only species producing a blue pigment and was considered to be distributed worldwide [2,19,20]. Three other species of blue *Haslea* were recently discovered, and formally described, including *Haslea karagadensis* Davidovich, Gastineau and Mouget [19] in the Black Sea, *Haslea provincialis* Gastineau, Hansen and Mouget [21] on the French coast of the Mediterranean Sea, and *Haslea nusantara* Mouget, Gastineau and Syakti [22] in the Java Sea. *Haslea karadagensis*, *H. provincialis,* and *H. nusantara* are morphologically similar to *H. ostrearia*. Although these four species are easily confused based on light microscopy (LM), they show significant differences in stria densities that are only visible using scanning electron microscopy (SEM) [19,21,22]. Furthermore, they are unambiguously distinguished by molecular barcoding and phylogeny [19,21,22]. Interbreeding experiments were unsuccessful at triggering auxosporulation between strains of these blue *Haslea* species, suggesting they belong to different taxa according to the concept of biological species [19,21,23].

In 2009, we observed blue diatoms in water samples from La Gomera, an island from the Canary Archipelago. Based on their morphology, they appeared to belong to a new species thereafter called *Haslea silbo* sp. nov. Several subsequent findings suggested that this species is a cosmopolitan taxon found in different geographic locations on both sides of the Atlantic Ocean. Preliminary characterization of this species and its blue pigment were done in the past [3], without the formal description being provided. Here, we present data collected during a decade of research on *Haslea silbo* sp. nov. These studies include the formal description of this new species, physico-chemical analyses of the blue pigment, evidence for auxosporulation, a molecular phylogeny as well as a genomic comparison of two sub-populations based on the sequencing of complete organellar genomes.

## 2. Materials and Methods

### 2.1. Isolation and Culture of Algae

Water samples were collected from the Canary Islands (Spain) at San Sebastián de La Gomera (28°05′ N; 17°06′ W) on February 2009. One mL of water sample and 0.5 mL L1 medium [24] were added to each well of a 24-well tissue culture plate (Sarstedt Inc., Newton, MA, USA). Growth conditions were 20 ± 1 °C, irradiance of ca. 35 µmol photons m^−2^ s^−1^, 12 h:12 h L:D cycle. After ca. two weeks of incubation under these conditions, blue *Haslea silbo* sp. nov. flourished in one well, and several monoclonal cultures (K-1283 to K-1289) were established by single cell micro-pipetting.

Blue diatoms morphologically similar to the strains from La Gomera were later sampled in North Carolina (USA) in July 2013, and thereafter isolated and kept in the Nantes Culture Collection (NCC) with the references NCC 454, NCC 455, and NCC 456. Other strains were isolated from the Gulf of Naples (Italy) between November 2014 and May 2015 at the LTER-Marechiara Station (40°48′ N, 14°15′ E) for strains NaS3, NaS23, NaS31, NaS32, NaS33, NaS34, NaS35, in the South Adriatic Sea in November 2016 (42°32′N, 17°59′E) for strain BIOTAII-43, and in Mersin, Turkey (36°47′ N, 34°35′ E) on March 2017 for strain SZCZMV2009.

Cultivation of *H. silbo* strains was conducted in 250 mL Erlenmeyer flasks containing 150 mL of modified artificial sea water (ASW) [25], under controlled conditions (15 ± 1 °C, irradiance of 60 µmol photons m^−2^ s^−1^, 14 h:10 h L:D cycle) at Le Mans Université. Culture illumination was provided by fluorescent tubes (Philips TLD 36W/965) and irradiance was measured using a Li-Cor LI-189 quantum meter coupled with a 2Π Li-Cor Q21284 quantum sensor.

### 2.2. Microscopy

Different protocols were employed for scanning electron microscopy (SEM) analyses, depending on the strains studied and the institution where the study was conducted. The original material from La Gomera was cleaned of organic matter according to Hendey [26], except that cells were collected by centrifugation rather than sedimentation during repetitive washings (3–4 times) in distilled water. Cleaned cells were mounted on glass slides with Naphrax and observed in LM using an Olympus BX51 (Tokyo, Japan), with a DP72 digital camera, in differential interference contrast and phase contrast. For SEM examinations, cleaned frustules were air-dried onto circular coverslip mounted on an aluminum stub and sputter coated with either gold, platinum, or palladium-platinum alloy. Observations were done, depending on the facilities, using a JUC5000 or JEOL JFC2300 HR High Resolution Fine and JEOL 7600F or JEOL J 6335F or JEOL JSM-5600 (JEOL, Tokyo, Japan) or FEG Tescan MIRA3 microscope (Brno, Czech Republic). For TEM material was air-dried onto 200 mesh Formvar covered copper grid. The TEM observations were made using a JEOL JEM-1010 (Tokyo, Japan) operated at 80 kV, equipped with a GATAN Orius SC1000 digital camera (Pleasanton, CA, USA). Measurements of valve structural elements were made from SEM images using the ImageJ software (http://rsb/info/nih/gov/ij (accessed on 1 September 2010)).

### 2.3. Pigment Extraction and Purification

Strain K-1285 was maintained in exponential growth phase and the biomass was collected every week. Cells and supernatant were separated by gentle centrifugation (10 min, 900 g, slow acceleration/deceleration, 4 °C), using a Sigma 3K15 and a rotor n°11133 (Bioblock Scientific, Illkirch-Graffenstaden, France). Pigments were purified and quantified according to the method developed by Pouvreau et al. (2006) [27], which consists of two-step ultrafiltration and anion-exchange chromatography.

### 2.4. UV–Visible Spectrophotometry

Purified pigment from *H. silbo*, intracellular and extracellular forms, were dissolved in distilled water at a concentration of 10 mg mL^−1^. A series of flasks containing 100 mL of 0.25 M Na_2_HPO_4_/NaH_2_PO_4_ buffer were prepared, with pH ranging from 2 to 12. The different buffers were filtered on a 0.22 µm membrane using a syringe. A mixture of 50 µL of buffer and 50 µL of pigment solution was placed in a Hellma quartz cell (Hellma, Germany), and homogenized by shaking using parafilm as seal. Absorbance measurements were done using a Thermo electron Helios gamma UV–visible spectrophotometer (Cambridge, UK). Blank measurement was made with buffer solution and spectra were recorded from 220 nm to 800 nm.

### 2.5. Raman Spectrometry

In vitro Raman spectrometry was performed as described previously [19,21] using purified marennine from *H. ostrearia* as a reference. Then, 200–500 µg of both intracellular and extracellular forms of *H. ostrearia* and *H. silbo* pigments were dissolved in 0.25 M Na_2_HPO_4_/NaH_2_PO_4_ buffer, pH 7, and the mixture was then placed on a glass slide and dried at room temperature. The laser beam was focused on the dry aggregates on the slides, on the darkest areas, where pigments were at seemingly higher concentration. Raman spectra were collected from different areas of the sample and averaged with an integration time varying between 200 and 600 s and an excitation power below 0.3 mW. For each series of measurements, the 514.5 nm wavelength radiation from a coherent spectrum argon/krypton ion laser built by Coherent Innova (Utrecht, the Netherlands) was selected to provide a good signal/noise ratio. The Raman spectra were recorded in the back-scattering configuration on a confocal T64000 Horiba, Jobin Yvon spectrometer (Longjumeau, France) coupled to a liquid-N2-cooled Charged Coupled Device detector. All Raman spectra were recorded within the 250–2250 cm^−1^ wavelength region and were shifted in the figures (with no baseline correction) for clarity.

### 2.6. Induction of Auxosporulation and Reproductive Behaviour

Monoclonal cultures were maintained in exponential growth phase by semi-continuous cultivation and periodic dilution with fresh medium, under the conditions described above. Cell density of cultures was estimated using a Nageotte counting cell (Hecht, Germany). Mixtures of parental cultures were made in sterile polystyrene Petri dishes at a concentration of 2000 cells mL^−1^. The first set of experiments was conducted using one pair of sexually compatible clones of *H. ostrearia* (NCC158.4 and NCC148.78), three clones of *H. silbo* (K-1283, K-1286, and K-1287), and one clone of *H. karadagensis* (NCC313). Clones were crossed pairwise. Culture plates with mixed parent clones were sealed with parafilm to prevent desiccation and placed in a growth chamber under a 6 h:18 h L:D cycle, with an irradiance ≤30 µmol photons m^−2^ s^−1^. Experiments were run in triplicate. The second set of experiments was conducted later using monoclonal cultures of *H. silbo* originating from the Gulf of Naples. In this case, the mating protocol and culture conditions were similar to those employed with *H. karadagensis* [23].

### 2.7. Molecular Barcoding

Molecular barcoding was performed on the original strains of *H. silbo* from la Gomera in the same way as performed on *H. karadagensis* and *H. provincialis* [19,21]. A similar protocol was used to amplify the *rbcL* gene of strain NaS3 from Naples. For the South Adriatic strain BIOTAII-43, DNA was isolated from 50 mL of an exponentially growing culture using the DNeasy Plant Mini Kit (Qiagen, Hilden, Germany). The purity of the extracted DNA was assessed with the NanoDrop spectrophotometer (BioSpec-nano Shimadzu, Kyoto, Japan). The nuclear 18S (SSU) rRNA gene and two chloroplast-encoded genes (*rbcL*, *psbC*) were amplified using the EmeraldAmpMax PCR Master Mix© (Takara Bio, Mountain View, CA, USA). When necessary, nested PCR reaction was done, with the PCR product from the first reaction used as a template for second reaction. The PCR products were visualized in a 1% agarose gel and then purified with StartaPrep PCR Purification Kit (Agilent Technologies, Inc., Santa Clara, CA, USA). The purified products were sent for Sanger sequencing (Macrogen©, Amsterdam, The Netherlands). All sequences were checked, edited, and assembled (5′–3′ and 3′–5′ ends) using Sequencher 4.1.4. In the case of NCC 456 and SZCZMV2009, molecular barcodes have been obtained using next generation sequencing, as described below. All DNA sequences have been submitted to GenBank and obtained registration number.

### 2.8. Next Generation Sequencing

Clones NCC456 and SZCZMV2009 were cultivated under the conditions described above. DNA was extracted following Doyle and Doyle (1990) [28]. Sequencing was conducted at the Beijing Genomic Institute Shenzhen facilities (Hong Kong, China). NCC456 was sequenced in 2015 on an Illumina Hiseq 4000 (22 million 150 bp paired-end reads, 250 bp insert) and SZCZMV2009 was sequenced in 2019 on a BGISEQ-500 (60 million 100 bp paired-end reads). Data were assembled using SPAdes 3.14.0 [29] with a k-mer of 85, and verified with Consed [30]. ORFs and genes encoded in the organelle genomes and plasmids were identified as previously described [31] and tRNA genes were localized with tRNAscan-SE 1.23 [32]. GenBank files were generated using Sequin 13.70 and were used to create maps using OGDRAW [33]. Whole genome alignments were obtained from progressiveMauve [34].

### 2.9. Phylogenetic Analysis

A maximum likelihood (ML) phylogeny was inferred from the partial *rbcL* gene using sequences obtained from PCR amplifications, extracted from complete plastid genomes or downloaded from GenBank. The *rbcL* sequences of the Naviculaceae *Seminavis robusta* Danielidis and Mann and *Trachyneis* sp., were added as outgroups. Sequences were aligned using MAFFT [35] and trimmed to a final length of 597 bp. The phylogenetic analysis was conducted using RAxML 8.0 [36] and data partitioned by first and second codon positions. The best tree out of 100 was computed for 1000 bootstrap replicates.

## 3. Results

### 3.1. Description

*Haslea silbo* Gastineau, Hansen and Mouget sp. nov.

Figures 1–17

**Figure 1:**
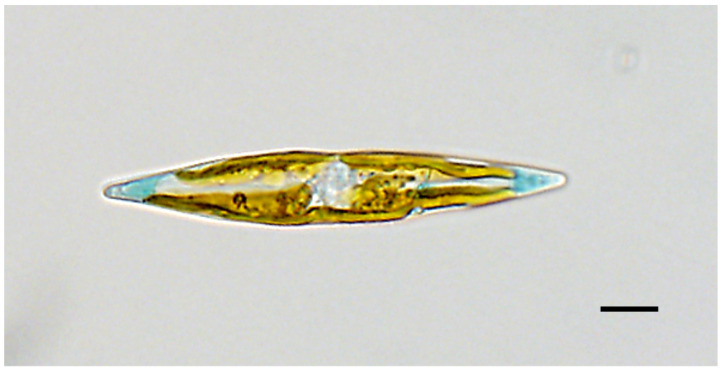
Live light microscopy (LM) image of *Haslea silbo* (valve view) clone K-1285. Scale bar 10 µm.

Description: In LM, cells are solitary, motile and strictly lanceolate with distinctly blue sub-acute apices (Figure 1). The pigmentation appears blue in cell apical vacuoles and green outside the cells when released in growth medium. Two band-like chloroplasts lie appressed to the girdle of the cell.

**Figure 2:**
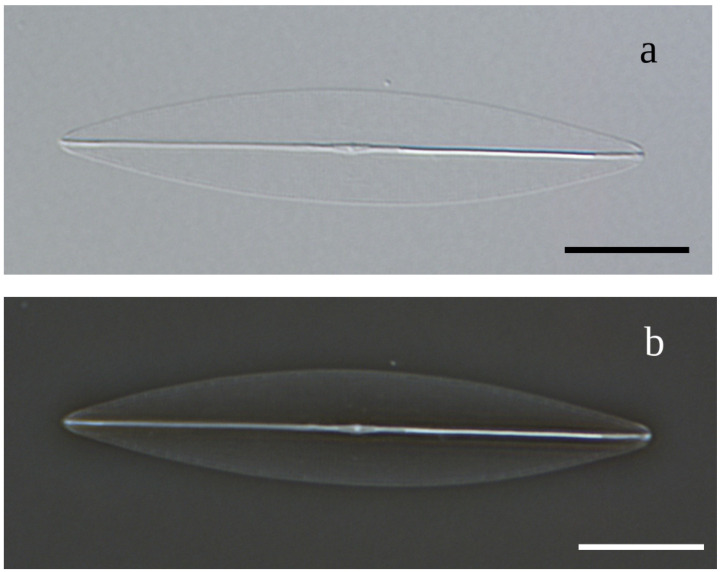
(**a**): LM-DIC (Light microscopy Differential interference contrast) image of cleaned frustules of *Haslea silbo* clone K-1285. (**b**): LM-PC (Light microscopy Phase-contrast) image of cleaned frustules of *Haslea silbo* clone K-1285. Scale bar 10 µm.

With cleaned frustules, only the valve outline and a distinct and straight raphe sternum are visible (Figure 2a,b). Length ranges between 138.0 µm for the largest initial cells to 20.2 µm for final stage cultures, with the corresponding widths of 10.0 to 5.0, respectively. At the time clone K-1285 was deposited as holotype at the Natural History Museum of Denmark, its length was 48.2 µm to 52.4 µm, and width 8.1 µm to 8.8 µm (n = 10).

**Figure 3:**
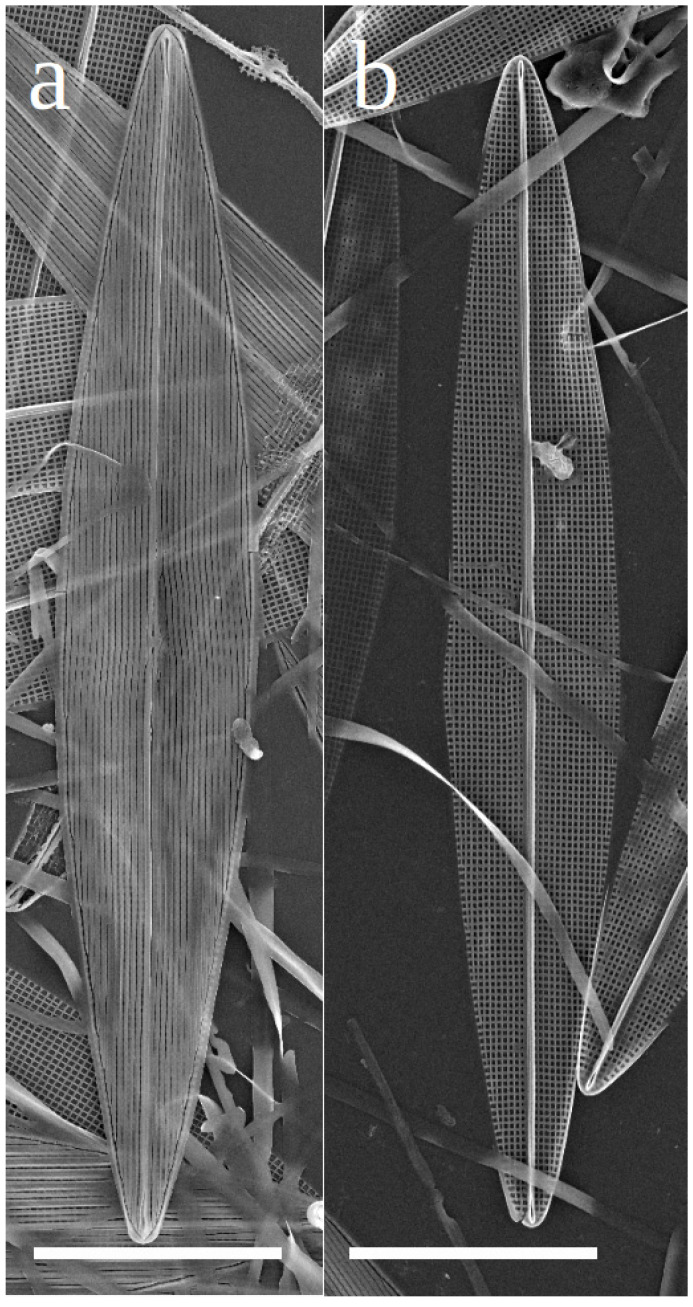
(**a**): Scanning electron microscopy (SEM) image of complete valves of *Haslea silbo* clone K-1285 in external view. (**b**): SEM image of complete valves of *Haslea silbo* clone K-1285 in internal view. Scale bars 10 µm.

In SEM, the valves are lanceolate with sub-acute apices (Figure 3a,b).

**Figure 4:**
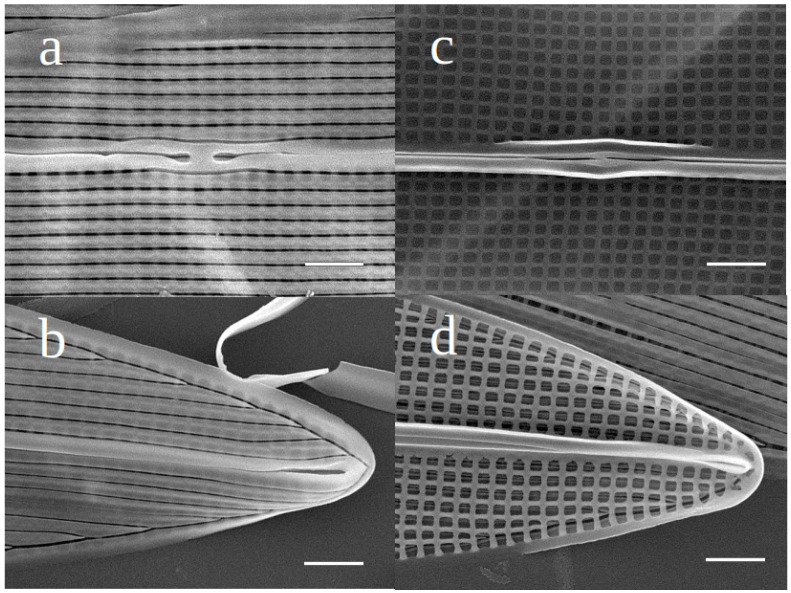
(**a**) SEM image of *Haslea silbo* clone K-1285, external view of the central raphe ending and longitudinal slits. (**b**) SEM image of *Haslea silbo* clone K-1285, external view of the straight apical raphe ending. (**c**) SEM image of *Haslea silbo* clone K-1285, internal view of the central ending of the raphe, notice the squarish areolae and the thin central bar. (**d**) SEM image of *Haslea silbo* clone K-1285, internal view of the apices with the straight apical ending of the raphe (Figure 9). Scale bar 1 μm.

Externally, the raphe fissures open slightly laterally in a narrow raphe sternum, ending more centrally (co-axially), with expanded closely-positioned ends. There is no lateral expansion of a central area. The apical raphe endings are straight, slightly expanded for the last micron (Figure 3a and Figure 4a,b). Internally the raphe fissures are straight, opening slightly laterally in the raphe sternum except at the center, where they are central in the very slightly widened sternum. At the poles the fissures terminate in narrow, slightly elevated helictoglossae. The raphe sternum is flanked by a very narrow accessory costa throughout its length on one side, with a short fine central bar on the other side (Figure 4c). Neither hide the raphe sternum (Figure 3b and Figure 4c,d). Externally the areolae open within narrow slits that run parallel to the raphe system throughout the valve, abutting the peripheral slits at the valve margins; the peripheral slits abut each other sharply at each apex (Figure 3a and Figure 4a,b). Internally the areolae are square (near center) to rectangular (near apices and margins), formed by the perpendicular intersection of the virgae (between transverse striae) and vimines (between longitudinal striae) (Figure 3b and Figure 4c,d). There are 32 transapical and 37–38 longitudinal striae in 10 µm (n = 10).

#### 3.1.1. Additional Morphometric and Morphological Data

**Figure 5:**
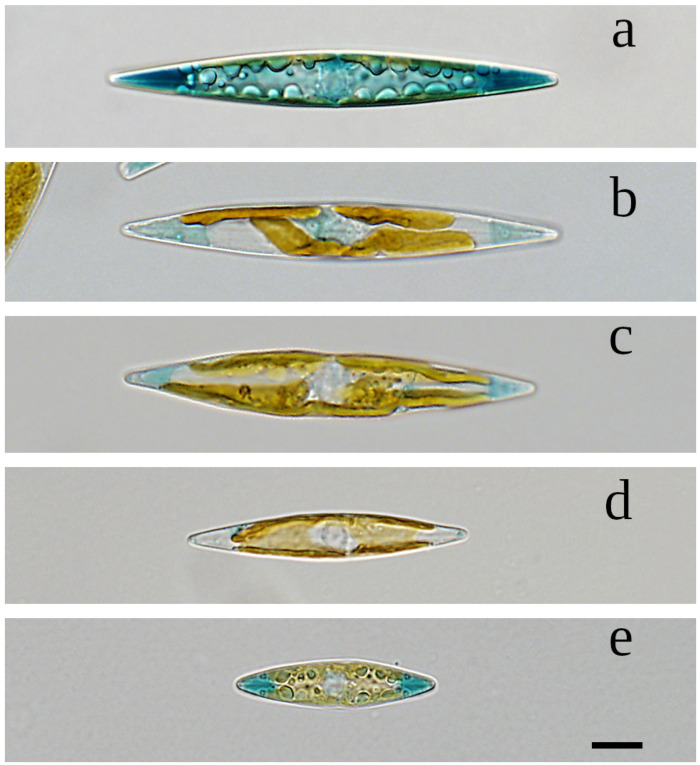
Reduction in cell size of *Haslea silbo* clone K-1285 between July 2009 and January 2012. Scale bar 10 µm. (**a**) July 2009. (**b**) February 2010. (**c**) November 2010. (**d**) August 2011. (**e**) January 2012.

Cell size gradually diminished in monoclonal cultures, with the length/width ratio also decreasing. Figure 5a–e illustrate this reduction in cell size because of the MacDonald-Pfitzer rule for the clone K-1285 between July 2009 and January 2012. The differences in the colour of the apices reflects the production of the marennine-like pigment, which seems triggered by higher light conditions.

**Figure 6:**
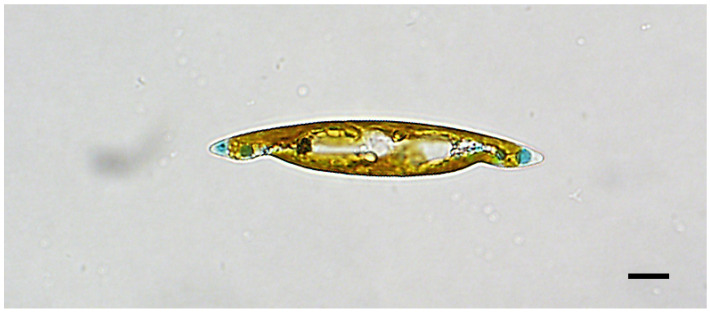
LM live image of a specimen of *Haslea silbo* showing deformities after months in culture. Scale bar 10 µm.

**Figure 7:**
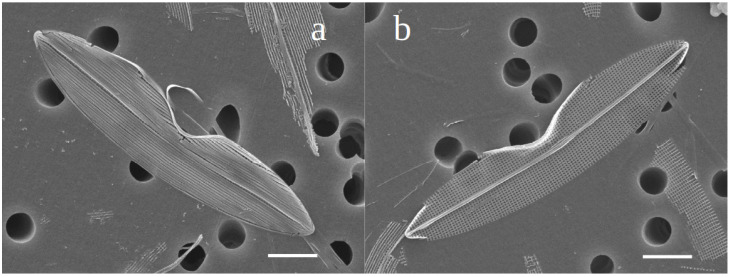
(**a**) SEM images of specimens of *Haslea silbo* showing deformities after months in culture in external view. (**b**) SEM images of specimens of *Haslea silbo* showing deformities after months in culture in internal view. Scale bar 5 µm.

As size diminished, frustules often exhibited deformities, as seen in LM on Figure 6 picturing (clone K-1285) and SEM on Figure 7a,b (clone K-1289).

#### 3.1.2. Holotype

Accession numbers CAT 2472 and CAT 2473 for a permanent slide of the K-1285 clone and an acid-cleaned sample of K-1285 kept in 70% ethanol, respectively, deposited at the Natural History Museum of Denmark, University of Copenhagen. Some valve parts of this type material are shown in Figure 2, Figure 3 and Figure 4.

#### 3.1.3. Type Locality

La Gomera, Canary Islands, Spain (28°05′26″ N; 17°06′54″ W), benthos.

#### 3.1.4. Etymology

The specific epithet silbo comes from the name of a whistled language “spoken” by the Guanches, native inhabitants of Berber origin of the Canary Islands, and still in use today in La Gomera.

#### 3.1.5. Ecology

Benthic species, found on beaches and as biofilm on rocks. Sometimes epiphyte on *Padina* spp.

#### 3.1.6. Molecular Signature

The ITS1-5.8S-ITS2 sequences are available in Genbank as HE663060 (K-1283) and HE978358 (K-1289). The *rbcL* sequences are available as HE663065 (K-1283), HE978358 (K-1289) (Canary Islands), MH355570 (NaS3), and MG189641 (BIOTAII-43). *SSU* and *psbC* partial sequences have also been obtained on BIOTAII-43 and registered as MG189639 and MG189640, respectively. For NCC456 and SZCZMV2009, complete organellar genomes and the cluster of nuclear rRNA genes were deposited in GenBank under accession numbers MW645081 (mitogenome NCC456), MW645082 (plastid genome NCC456), MW645083 (mitogenome SZCZMV2009), MW645084 (plastid genome SZCZMV2009), MW679567 (rRNA NCC456), and MW679566 (rRNA SZCZMV2009).

#### 3.1.7. Differential Diagnosis

*Haslea silbo* is very similar to *H. ostrearia*, *H. karadagensis* [19], *H. provincialis* [21], and *H. nusantara* [22], with respect to overall shape of the valves and the presence of blue apices. However, several characters differentiate these species. The apices in *H. silbo* appear blunter than in other blue *Haslea* species. *H. silbo* and *H. nusantara* share a thin central bar that discriminates them from the other species. The color of the blue apices may also help distinguishing *H. silbo* from *H. karadagensis*, whose apices appear darker and slightly grey. The most decisive feature is the stria density, which is lower in *H. silbo* compared to all other blue *Haslea* due to a significant reduction in the number of longitudinal striae, and the areolae are quadrate rather than rectangular.

### 3.2. Spectrophotometric Analyses of H. silbo’s Blue Pigment

**Figure 8:**
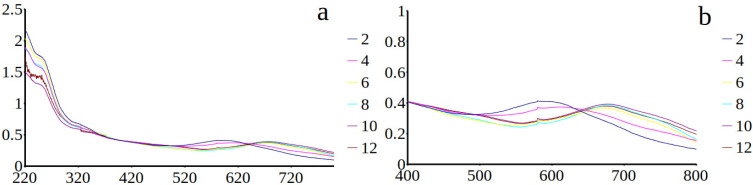
(**a**) Absorbance spectra from 220 to 800 nm of the purified intracellular form of the marennine-like pigment produced by *Haslea silbo* depending on pH ranging from 2 to 12 (inset on the right = colour of the curve/pH). (**b**) focus on the isobestic point in the visible region. *X*-axis = wavelength (nm). *Y*-axis = optical density.

**Figure 9:**
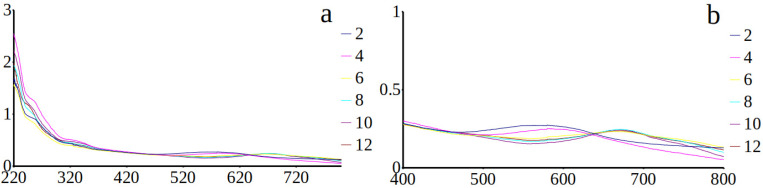
(**a**) Absorbance spectra from 220 to 800 nm of the purified extracellular form of the marennine-like pigment produced by *Haslea silbo* depending on pH ranging from 2 to 12 (inset on the right = colour of the curve/pH). (**b**) focus on the isobestic point in the visible region. *X*-axis = wavelength (nm). *Y*-axis = optical density.

UV–visible spectra obtained on intracellular (Figure 8a,b) and extracellular (Figure 9a,b) forms of the blue pigment produced by *H. silbo* were very similar and all displayed an isobestic point in the visible region of the spectrum at ca. 640 nm (Figure 9a,b). The position of the isobestic point at 640 nm is identical to what was already observed on the pigments of *H. provincialis* [21].

**Figure 10:**
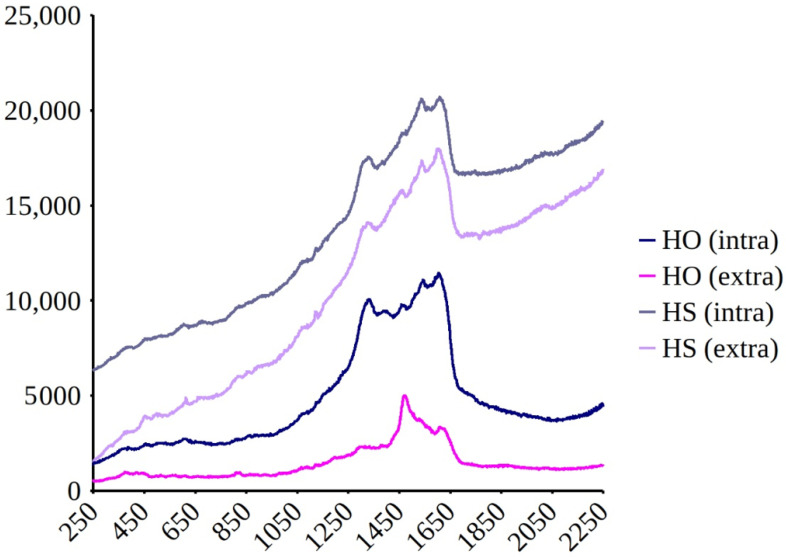
Comparison of the in vitro Raman spectra at 514.5 nm recorded on purified blue pigments of *Haslea ostrearia* (HO) and *Haslea silbo* (HS), intracellular form (intra) and extracellular form (extra). *X*-axis = Wavenumber (cm^−1^). *Y*-axis=Raman Intensity (arbitrary units).

Raman spectrometry (in vitro) of the intracellular and extracellular forms of purified *H. silbo*’s pigments showed similar spectra (Figure 10). The pigment of *H. silbo* seems similar to the intracellular marennine of *H. ostrearia* but obviously different when compared to the extracellular marennine.

### 3.3. Reproductive Behaviour

**Figure 11:**
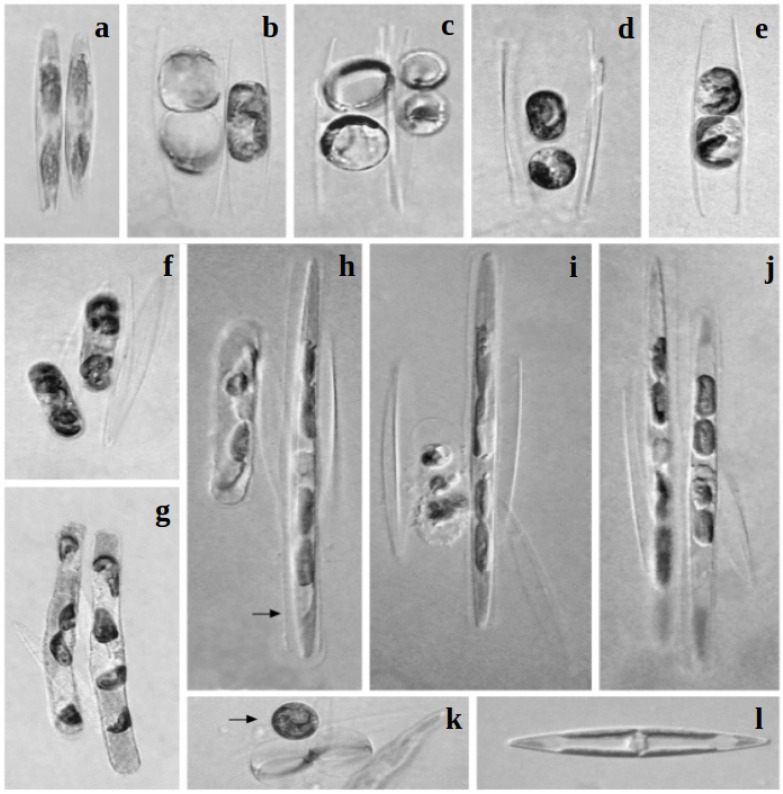
Stages of sexual reproduction in *Haslea silbo* (×400 magnification). (**a**) A pair of sexually compatible gametangial cells. (**b**) Gametes have been formed and swelled in the left and are still to be formed in the right gametangial cell. (**c**) Two gametes have been formed in each gametangial cell and are ready to fuse. (**d**) Two zygotes resulted from gamete fusion. Note two opened gametangial frustules (four thecae). (**e**) A pair of gametes in a separately lying gametangial cell may be confusingly understood as a pair of zygotes, but note that only two thecae are present in this case. (**f**,**g**) Growing auxospores. (**h**) Initial cell (right) formed in the envelope of the fully grown auxospore (arrow); development of the next auxospore is tardy. (**i**) One initial cell and destroyed auxospore. (**j**) Two normally developed initial cells. (**k**) A zygote (arrow) and two aborted gametes (below), swelled due to osmotic pressure. (**l**) A vegetative cell.

Intraclonal reproduction was absent in the control monoclonal Petri dishes and sexualization did not occur between any of the original *H. silbo* clones from la Gomera (K-1283 to K-1289). Sex-interaction was also absent between sexually competent *H. ostrearia* strains and *H. silbo* strains, or between *H. silbo*, *H. karadagensis* or *H. provincialis* strains. However, heterothallic reproduction was detected among *H. silbo* strains from the Gulf of Naples: clone NaS32 proved to be sexually compatible with clones NaS31, NaS34, and NaS35. The general pattern of auxosporulation (Figure 11) appeared to be similar to those previously observed for *H. ostrearia* [37,38], *H. karadagensis* [23], and *H. provincialis* [21]. The minimum and maximum sizes of the gametangia were 61 µm and 75 µm, respectively (n = 36). The minimum and maximum sizes of the initial cells were 115 µm and 138 µm, respectively (n = 36).

### 3.4. Genomics

#### 3.4.1. NCC456 Mitochondrial Genome

**Figure 12:**
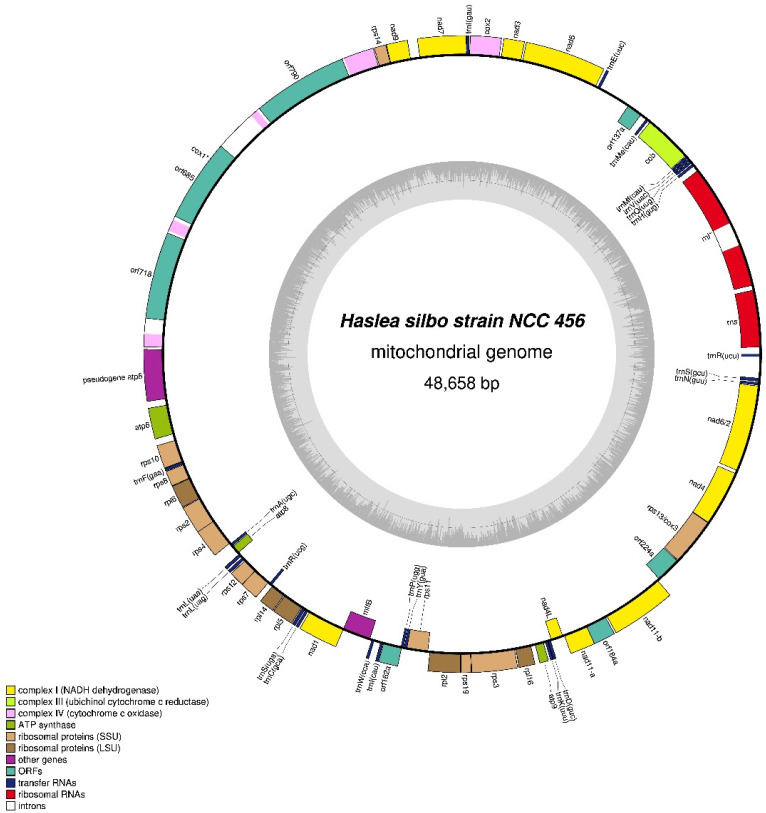
Map of the mitochondrial genomes of *Haslea silbo* NCC456.

The 48,658 base pairs (bp) mitochondrial genome of strain NCC456 encodes 58 conserved genes (34 protein-coding, 2 rRNA genes and 22 tRNA genes) (Figure 12). Two pairs of protein-coding genes are fused in single reading frames, namely, *nad6*/*nad2* and *rps13*/*cox3*. An *atp6* pseudogene of 1283 bp lies between *cox1* and *atp6*. The NCC456 mitochondrial genome also displays a conserved open reading frame of 162 codons (orf162) showing similarity with the orf147 of *Berkeleya fennica* Juhlin-Dannfelt (YP_009115295) [39,40]. This hypothetical gene is located in a conserved gene cluster extending from *tatC* (*mttB*) to *rps11*, which is found in several other diatom species [40]. There are also 3 other ORFs, namely, orf137a, orf184a, and orf224a. There are three group II introns in *cox1* that each contain an ORF encoding a putative reverse transcriptase (RT) (orf685, orf718, and orf790) displaying similarity with mitochondrial intron-encoded RTs from other diatoms. Moreover, a group II intron of 649 bp interrupts the *rnl* gene.

#### 3.4.2. SZCZMV2009 Mitochondrial Genome

**Figure 13:**
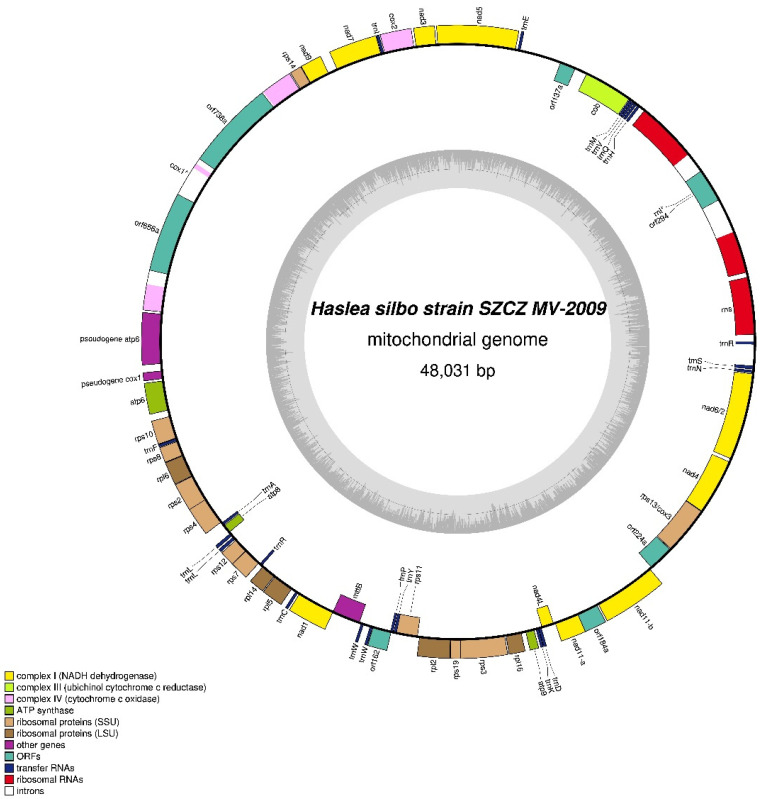
Map of the mitochondrial genomes of *Haslea silbo* SZCZMV2009.

The 48,031 bp mitochondrial genome of strain SZCZMV2009 encodes the same 58 conserved genes that are found in strain NCC456 (Figure 13) and also orf137a, orf184a, and orf224a. These genes display the same order in the two strains. Like the NCC456 genome, the SZCZMV2009 mitochondrial genome also contains the conserved orf162 as well as a 1283-bp *atp6* pseudogene. This pseudogene is located between *cox1* and a 210-bp region showing 99% identity with the 3′ end of cox1; the latter region is absent from strain NCC456. The *cox1* gene contains two group II introns, with different insertion points compared to the introns found in NCC456, that encode putative RTs (orf656 and orf738) showing similarity with mitochondrial intron-encoded RTs from other diatoms. The *rnl* gene is interrupted by a 2346-bp group II intron, at the same position as for the intron discovered in NCC456. This intron also encodes a RT (orf294).

#### 3.4.3. Comparison with The Mitochondrial Genome of *H. nusantara*

**Figure 14:**
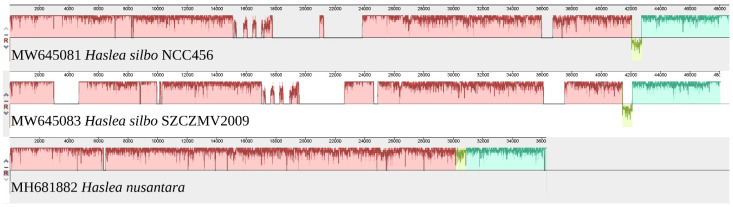
MAUVE alignment of the mitochondrial genomes of *Haslea silbo* NCC456 (MW645081), *Haslea silbo* SZCZMV2009 (MW645083), and *Haslea nusantara* (MH681882).

The whole genome alignment obtained from progressiveMauve is shown in Figure 14. The gaps observed when comparing *H. silbo* NCC456 and SZCZMV2009 represent the differences in intron contents, which also discriminates both of them from *H. nusantara*. The portion displayed in green corresponds to a change of strand of orf224 in *H. silbo* compared to the corresponding orf226 in *H. nusantara*. A comparison with the genome of *H. nusantara* (MH681882) and blastp analyzes of ist content showed that it also displays a fusion of *nad6*/*nad2*, although not indicated on GenBank. However, the fusion of *rps13*/*cox3* differentiates *H. silbo* from *H. nusantara*, in which both genes are distinct.

#### 3.4.4. NCC456 Plastid Genome

**Figure 15:**
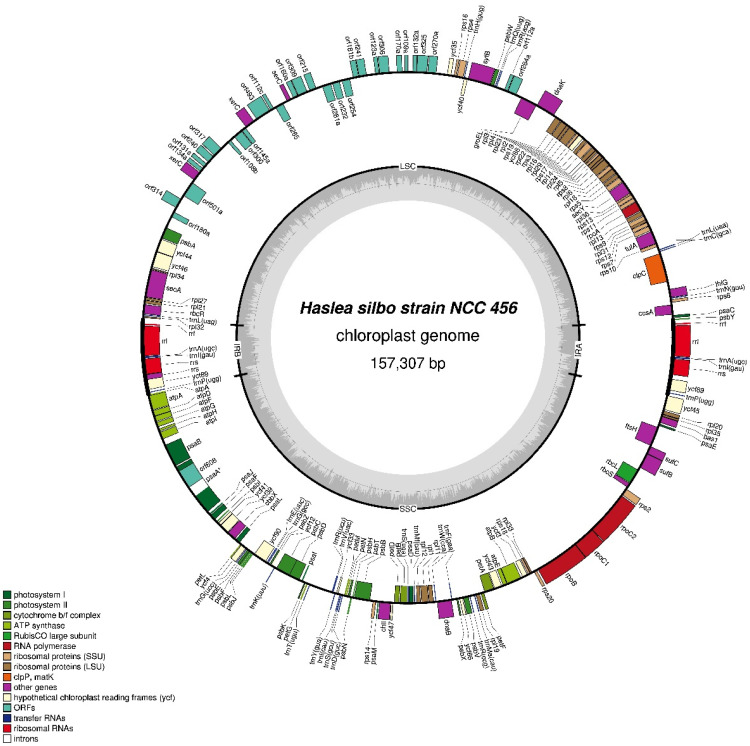
Map of the plastid genomes of *Haslea silbo* NCC456.

The 157,307-bp plastid genome of strain NCC456 displays an inverted repeat (IR) sequence of 7297 bp that separates this genome into large (LSC) and small (SSC) single-copy region of 75,060 and 67,653 bp, respectively (Figure 15). The LSC encodes for 51 conserved protein coding genes, 7 tRNA, but also displays 2 non-conserved ORFs comprised between *groEL* and *trnR*, and especially a large insertion of ca. 32,325 bp between *ycf35* and *psbA* that contains 28 non-conserved ORFs, two putative *xerC* genes coding for DNA integrases/recombinases and a putative *serC* gene coding for a serine recombinase. It is worth being underlined that the two putative *xerC* genes, although slightly differing in their lengths (888 bp and 939 bp), showed a remarkable identity of 79.21% as calculated by clustal omega. The SSC encodes for 74 conserved protein coding genes and 17 tRNA. A single group II intron of 2921 bp encoding a RT (orf608) is found in the *psaA* gene. This RT shows 83% sequence identity with the RT identified in the same gene of the diatom *Toxarium undulatum* Bailey [41]. The IR encodes for a single conserved protein coding gene, three rRNA genes, and three tRNA.

#### 3.4.5. SZCZMV2009 Plastid Genome

**Figure 16:**
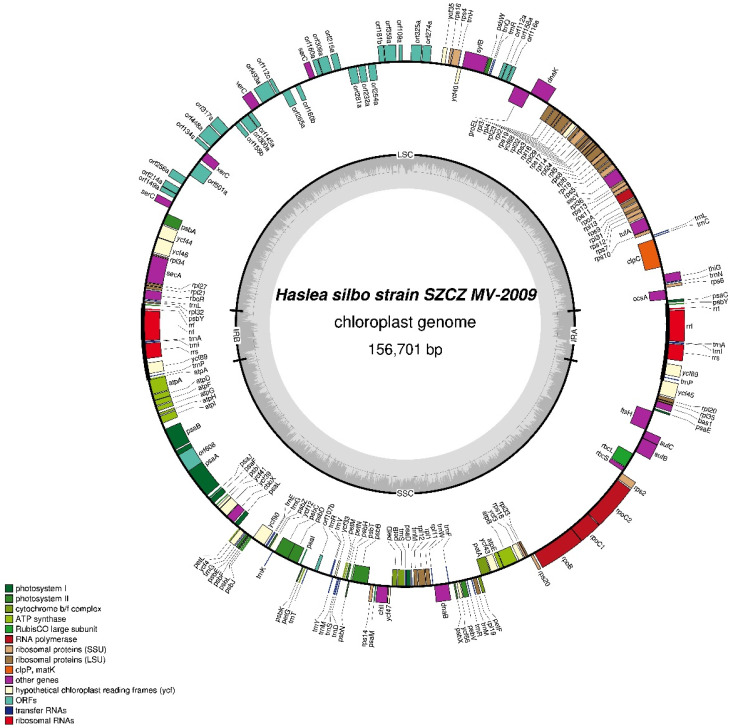
Map of the plastid genomes of *Haslea silbo* SZCZMV2009.

The 156,701 bp plastid genome of strain SZCZMV2009 (Figure 16) has the same gene content and gene order than the genome of strain NCC456. The LSC is 67,653 bp long, the SSC is 74,454 bp long and the IR is 7297 bp long. The insertion sequence between *ycf35* and *psbA*, which is slightly shorter (31,719 bp) than the homologous region in strain NCC456, contains 25 non-conserved ORFs, two putative *xerC* genes and two putative *serC* genes. Furthermore, between *groEL* and *trnR* there are three ORFs instead of two when compared to NCC456. As in the NCC456 plastid genome, a 2921-bp group II intron encoding a RT (orf608) is present in *psaA*.

#### 3.4.6. Comparison with The Plastid Genome of *H. nusantara*

**Figure 17:**
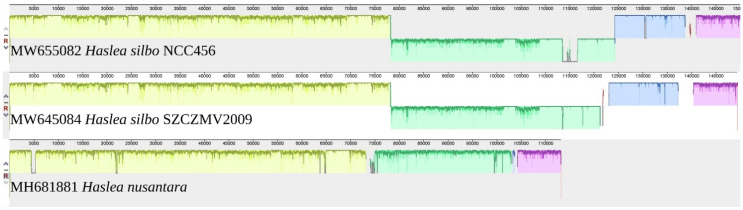
MAUVE alignment of the plastid genomes of *Haslea silbo* NCC456 (MW645082), *Haslea silbo* SZCZMV2009 (MW645084), and *Haslea nusantara* (MH681881).

The whole genome alignment obtained from progressiveMauve is shown in Figure 17. The region in emerald green represents a whole portion of the genome, located between *ycf35* and *clpC* and comprising the cluster of ribosomal protein genes, which has been rearranged, while the region in blue, which is nearly entirely lacking in *H. nusantara*, represents the region of insertion between *ycf35* and *psbA*.

#### 3.4.7. NCC456 Plasmids

Three circular plasmids, namely, pHSC1 (3534 bp; MW651861), pHSC2 (4440 bp; MW651862), and pHSC3 (4426 bp; MW651863), each featuring a putative serine recombinase gene (*serC*), were identified among the contigs of the NCC456 assembly. Their circular nature was deduced from the overlapping extremities displayed by each of these contigs. The best blastp results for these putative serine recombinase proteins are AZJ16760 from the plastid genome of the diatom *S. robusta* (evalue 3^e-117^, identity 77.3%), YP_003734627 from the plastid genome of the dinotom *Kryptoperidinium foliaceum* (F.Stein) Lindemann (evalue 4^e-119^, identity 79.62%), and NNL21571 from the bacteria *Ignavibacteriaceae bacterium* (evalue 5^e-121^, identity 80.66%) for pHSC1, pHSC2, and pHSC3, respectively. Plasmids also code for other hypothetical proteins, which are orf480 for pHSC1; orf361 and orf513 for pHSC2; orf138, orf350, and orf515 for pHSC3.

#### 3.4.8. SZCZMV2009 Plasmids

Three circular plasmids, namely, pHSM1 (3556 bp; MW651864), pHSM2 (4513 bp; MW651865), and pHSM3 (4117 bp; MW651866), were also identified among the contigs of the SZCZMV2009 assembly. Both the pHSM1 and pHSM3 plasmids are highly similar in sequence (99.86% and 99.56% identity) to the NCC456 pHSC1 and pHSC3 plasmids, respectively, as they also code for the same putative proteins and ORFs). On the other hand, the pHSM2 plasmid sequence appears to be specific to the SZCZMV2009 strain, as no homologous sequence was recovered among the contigs of the NCC456 strain. The plasmid sequence pHSM2 codes for a putative serine recombinase whose best blastp match is the already evoked AZJ16760 from *S. robusta* (evalue 3^e-126^, identity 80.73%).

#### 3.4.9. Phylogeny

**Figure 18:**
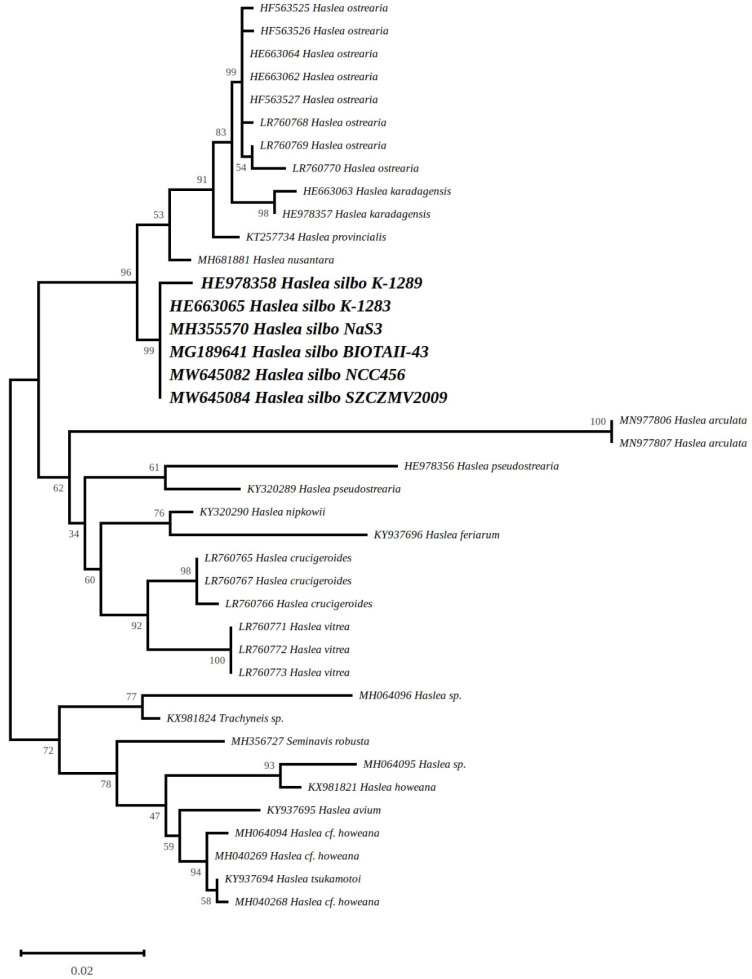
Maximum Likelihood tree obtained from partial *rbcL* genes. The best tree out of 100 (score −1860.998387) was computed for 1000 bootstrap replicates.

The tree inferred from the *rbcL* sequences of 40 diatoms displays a strongly supported clade (96% bootstrap support) containing all five *Haslea* species that are able to produce a blue pigment (*H. silbo*, *H. nusantara*, *H. provincialis*, *H. karadagensis,* and *H. ostrearia*) (Figure 18). *Haslea silbo* appears at the base of this clade. Apart from the clade of blue diatoms, two major clades can be observed in the tree. The clade sister to the blue *Haslea* clade contains non-blue species whose classification has reached a consensus (*Haslea pseudostrearia* Massé, Rincé and Cox, *Haslea nipkowii* (Meister) M.Poulin and G.Massé, *Haslea feriarum* M.A.Tiffany and F.A.S.Sterrenburg, *Haslea vitrea* (Cleve) Simonsen, *Haslea crucigeroides* (Hustedt) Simonsen, and *Haslea arculata* Lobban and Ashworth) [42,43,44,45]. The third major clade contains the species of pennate diatoms which were meant to be the outgroup (e.g., *Trachyneis* sp.) as well as numerous species listed as *Haslea* spp., but whose exact identities and classification have been recently linked to *Navicula sensu lato* [44].

## 4. Discussion

This study describes *Haslea silbo*, the fifth species of blue *Haslea*, also the second whose cosmopolitanism is demonstrated by molecular methods, raising questions about biodiversity, geographical distribution, and phylogeny as well as endemism/cosmopolitanism among benthic diatoms. These findings also illustrate the general trend of increasing number of cryptic or pseudo-cryptic species of diatoms discovered, made possible by the availability of supplementary and complementary tools for taxonomical studies, including electron microscopy, mating experiments, and molecular biology [46,47,48,49,50,51,52,53]. Most of these studies underscore the necessity of using a combination of methods for reliable identification at species level.

*Haslea ostrearia* was the first blue diatom to be described [2], but the assumption of its cosmopolitan distribution goes back to the times when only LM was available. It is now certain, as it was demonstrated by SEM and molecular taxonomy, that *H. ostrearia* can be found in both hemispheres, as well as in the Atlantic and Pacific Ocean [20,54]. On the contrary, *H. karadagensis*, *H. provincialis,* and *H. nusantara* seem to have a limited distribution, having only been observed in the Black Sea, the Mediterranean Sea, and the Java Sea, respectively. In contrast, *H. silbo* sp. nov. has been identified in multiple locations that are far from each other (the Canary archipelago, the North Carolina coasts, and three different sites of the Mediterranean Sea). This species might have been observed on the shores of Brazil [55], but its identity was not confirmed by molecular taxonomy.

Morphologically, *H. silbo* is very similar to other blue *Haslea* species, *H. ostrearia*, *H. karadagensis*, *H. provincialis*, and *H. nusantara*. However, the morphometrics showed significant differences regarding the density of the longitudinal striae, which is lower in *H. silbo*, and also regarding the size and shape of the areolae, which are larger and more quadratic, respectively. Moreover, like *H. nusantara*, *H. silbo* displays a central small thin bar on the internal surface of its valve, but there is none in the other blue species described yet [22]. This shared character between *H. silbo* and *H. nusantara* may be of some taxonomic significance, considering the close position of these two species in the phylogeny inferred from *rbcL* sequences. As is the case for *H. provincialis* [21], *H. silbo* seems to possess a single form of the marennine pigment, differently from *H. ostrearia* for which two different forms, intracellular and extracellular, have been described.

The process of sexual reproduction appears to be very conserved within the genus *Haslea*, as it was observed among either blue [21,23,37,38] or non-blue species [56,57]. At 138 µm, the maximum size of the initial cells observed for *H. silbo* appeared to be higher than the values measured for *H. karadagensis* and *H. provincialis* (97 µm and 109 µm, respectively) but lower than the 143 µm measured for *H. ostrearia* [21,23,37,38]. The unsuccessful mating experiments conducted on the strains from La Gomera might have been the result of having only one mating type among these strains, as it was shown in similar studies with other *Haslea* or *Pseudo-nitzschia* species [37,46]. Note here that the former experiments were conducted with only three clones of *H. silbo*, which is the strict minimal number of clones needed to most likely have two compatible mating types according to Chepurnov et al. (2004) [58]. Furthermore, there is a possibility that all the monoclonal cultures from La Gomera might have as well correspond to a single individual, as they were established from the same enrichment micro-well, inoculated with only 1 mL of sample. Unfortunately, the clones from la Gomera died before it was possible to perform mating experiments between them and the strains from Naples.

When the first *H. silbo* strains were discovered a decade ago, little was known about the distribution and diversity of blue diatoms, which led to consider it might be an endemic species from the Canary Islands. Few studies have been reported on the diatom flora of the Canary Islands [59,60,61,62], and only one of them refers to the presence of *H. ostrearia*, along with *Haslea britannica* (Hustedt and Aleem) Witkowski, Lange-Bertalot and Metzeltin [63], without providing information about the island concerned. *Haslea spicula* (Hickie) Bukhtiyarova was also listed [64]. Cleaned frustules of *H. britannica* and *H. spicula* could hardly have been misidentified with *H. silbo*, because of obvious differences in densities of striae (for both species) or the presence of a pseudo-stauros (a thickening of its central costa detectable in light microscopy) in *H. spicula* [65].

The Canary Islands are both geographically isolated and at the confluence of important ocean shipping routes, which raises a paradox concerning the geographical origin of the *H. silbo* isolates identified there. These islands form an archipelago composed of volcanic islands, located ca. 100 km north-west of the African coast but geologically distinct from the African continental shelf. The distance between neighboring islands ranges between ca. 20 km to ca. 100 km. First mention of the Canary Islands can be ascribed historically to the Phoenicians. The islands were successively visited and/or conquered by the ancient Greeks and Romans, then the Spanish during the fifteenth century. The Canary Islands were thus used as port of call for Columbus’ expeditions. After 1492, Las Palmas de Gran Canaria and Santa Cruz de Tenerife were common stopping points for ships going to, and coming from the New World. Similarly, the long history of the port of Naples, founded in the ninth century BC by Greek settlers, is in accordance with the hypothesis of human-mediated transfer of species, Naples having belonged to several maritime empires, like Byzantium, Normans, and Spain during the Spanish golden century.

As we demonstrated that *H. silbo* is a cosmopolitan species, it can be hypothesized that human-mediated translocation to or from the archipelago and other geographical locations happened. Such transfers could have been facilitated (or promoted) by ballast water [66,67]. Alternatively, non-human mediated transfers could come from sea mammals or sea turtles [68,69,70], drifting plastic [71], and from translocation of shellfish, e.g., due to the introduction of the European flat oyster in North America during the 1940s and 1950s [72,73]. Note here that oyster transfers have been documented as a vector for the introduction of other organisms [74].

Given the important role of the Canary Islands as ports of call, it is reasonable to hypothesize that the dispersal of *H. silbo* on both sides of the Atlantic Ocean has been human mediated. As soon as the early sixteenth century Spaniards went from Mediterranean Sea to what is today North Carolina, with stopover in the Canary Islands. The internal traffic in the Mediterranean Sea, between East and West, goes back in history as far as the history of the region itself, and both ways are of course still currently in use. This is the most coherent hypothesis that can be proposed regarding the dispersal of a benthic species, which is most likely to sink and form biofilms rather than drifting in the water column like a planktonic species and consequently, the true geographical origin of this taxon will probably remain a mystery.

The phylogenetic position of *H. silbo* seems to dispute with *H. nusantara* [22] a more basal position in the blue clade. Narrowing the gap between blue and non-blue species, especially to the cosmopolitan *H. pseudostrearia* [75], will be an important step for “omics” investigations about the mechanisms of appearance and development of the blue pigment biosynthetic pathway among the *Haslea* species.

Since the mitochondrial and plastid genomes of *H. nusantara* have already been described [22], it is possible to draw some comparisons between the two species. At 120,448 bp, the size of the *H. nusantara* plastid genome is significantly smaller than the sizes observed for *H. silbo*. The size of the *H. silbo* plastid genome is closer to those of *S. robusta* (150,905 bp) [76], *Eunotia bilunaris* (Ehrenberg) Schaarschmidt (152,906 bp) and *Cylindrotheca closterium* (Ehrenberg) Reimann and Lewin (165,809 bp) [77]. The extra length of the *H. silbo* plastid genome relative to that of *H. nusantara* is explained by the large insertion of DNA from a putative plasmidial origin between *psbA* and *ycf35*. The size of this insertion, as well as the number of putative serine recombinase and DNA integrase/recombinase genes in it, suggest that several plasmids were concerned. Similar insertions have been observed in the plastid genomes of *C. closterium* (KC509522) [77], *Asterionellopsis glacialis* (Castracane) Round (KC509520) [77], *S. robusta* (MH356727) [76] and the dinotom *K. foliaceum* (GU591328) [78]. Further, *serC* genes have been observed in the plastid genome of *Nanofrustulum shiloi* (J.J.Lee, Reimer and McEnery) Round, Hallsteinsen and Paasche (MN276191) [79]. *Haslea silbo* is the second diatom species after *T. undulatum*, a centric mediophycean diatom, to present a group II intron in its *psaA* [41]. Several circular plasmids, similar to those found in *Cylindrotheca* Rabenhorst [77,80,81], were discovered in the two *H. silbo* strains analyzed. All these plasmids, which are only known among pennate diatoms [82], bear a putative serine recombinase *serC* gene.

Concerning the mitochondrial genome, in contrast to species such as *Phaeodactylum tricornutum* Bohlin, *Thalassiosira pseudonana* Hasle and Heimdal [83], *Synedra acus* Kützing [84], or *Asterionella formosa* Hassall [85], there are no repeated regions in the *H. silbo* mitogenome, which is a situation also found in *H. nusantara* and *B. fennica* [39]. The mitogenome of *H. silbo* is very similar in size (48,031 bp and 48,658 bp) to that of *N. ramosissima* (C. Agardh) Cleve (48,652 bp) (KX343079) [85], mainly because of the presence of the three group II introns within the *cox1* gene. Interestingly, the intron orf685 is only loosely associated with introns discovered in diatom mitochondria (*Ulnaria acus* (Kützing) Aboal, e-value 9^e-86^, identity 32%) (GU002153) [84]. The intron in *rnl* displays similarities with the one present in the same mitochondrial gene of *N. ramosissima* [86].

Perhaps, the most intriguing features of the *H. silbo* mitogenome are the presence of an *atp6*-like pseudogene near the functional gene, which is conserved between NCC456 and SZCZMV2009, and the fusion of two pairs of protein coding genes. Nuclear pseudogenes of mitochondrial origin are well documented among animals [87], and mitochondrial pseudogenes kept in the mitochondrion have been discovered among various animals too [88,89,90,91,92,93,94]. In plants and algae, there are very few records of pseudogenes in mitochondria [95,96,97,98,99]. Regarding the fusion of genes inside single ORFs, the one concerning *cox3* and *rps13* is the most disturbing. Hence, while *nad2* and *nad6* code for sub-units of the same protein (NADH dehydrogenase), the biological functions of *rps13* and *cox3* are absolutely different. Thus, there might be an obligatory mechanism leading to the obtention of two distinct and functional proteins, which is yet to be discovered.

Furthermore, it is worth to note that regarding the *cox1* gene, alignments of the CDS for clones NCC456 and SZCZMV2009 showed a 99.53% identity between them. When trimmed and aligned to the 721 bp *cox1* fragments that were obtained on *H. ostrearia* [54] the identity rose to 99.58%, with only three polymorphisms. For the corresponding portion of gene of *H. ostrearia*, seven polymorphisms were found [54] and as far as our knowledge on these species goes, no *cox1* introns were evidenced in any of the populations of *H. ostrearia* studied up to now. A broader sequencing effort devoted to blue *Haslea* mitogenomes would be needed to draw any conclusion, however the results obtained on *H. silbo* may suggest that the dynamics of introns is not such an infrequent event when compared to the occurrence of SNPs.

## 5. Conclusions

The present study expands our knowledge on the biodiversity and biogeography of species from the genus *Haslea* producing blue pigments. We describe a new cosmopolitan species, *Haslea silbo* sp. nov., using a multi-pronged protocol. This protocol includes the use of next generation sequencing, which has proven to provide highly valuable results, both at the species and population levels. For the further fulfillment of our studies on blue diatoms, we already consider a systematic use of NGS for any description of new population and species.

## Data Availability

All molecular data have been deposited on GenBank has described above. Sequences have also been deposited on Zenodo with the following permanent link (http://doi.org/10.5281/zenodo.4588885 accessed on 8 March 2021).
